# Supplementary material for: Corals that survive repeated thermal stress show signs of selection and acclimatization
Source: PLoS One. 2024 Jul 31;19(7):e0303779. doi: 10.1371/journal.pone.0303779 (PMC11290665; doi:10.1371/journal.pone.0303779)
Supplement: S1 File — (DOCX) [file pone.0303779.s001.docx]

***Corals that survive repeated thermal stress show signs of selection and acclimatization***

**Supplemental information**

Quantifying heat stress

We quantified heat stress at each site using degree heating weeks (DHW), a metric of accumulated heat stress that has been shown to correspond well to coral bleaching thresholds [1,2]. Widespread coral bleaching is expected above 4 DHW, while widespread coral mortality is expected above 8 DHW [3–5]. DHW for each study site were obtained from NOAA’s Coral Reef Watch (CRW) program. DHW values were based on satellite-derived sea surface temperature (SST) data with a 5 km spatial resolution. Most sites were situated far enough apart that they fell into distinct 5 km cells, but two sites (Kahekili and Wahikuli) were close enough that they fell within the same 5 km grid cell and thus had the same DHW values. All sites had zero DHW at the time of sampling in 2014, 2017, and 2021, while sites ranged from 6.75 to 9.40 DHW when sampled in 2015 and from 7.80 to 9.61 DHW when sampled in 2019 (Figure S1, Table S1).

To corroborate DHW estimates, we compared SST values from the satellite-derived CRW data to SST values modeled using the Regional Ocean Modeling System (ROMS) for the Main Hawaiian Islands. ROMS forecasts sea water temperature at 4 km grid resolution for multiple depth strata, and the model assimilates both satellite and *in situ* ocean temperature data to improve forecasts [6]. We calculated the difference between SST values obtained from CRW and ROMS at each of our sites (Figure S2). SST tracked closely for the duration of both bleaching events, except for the very end of the 2019 bleaching event, where ROMS forecasts cooler SST values than CRW (Figure S2). This discrepancy began in November 2019, which is after our 2019 surveys and after DHW peaked for all sites. Thus, this temperature discrepancy should not explain reductions in bleaching observed in this study. However, if ROMS is picking up on a real drop in temperature that CRW missed, it could help to explain improved coral recovery following bleaching in 2019 compared to 2015.

Comparison of tracing accuracy between annotators across software platforms

Researchers in this study used two software platforms to trace patches of live coral tissue: TagLab and ArcGIS Pro. The commercially available ArcGIS Pro has a wide variety of geospatial applications, and is the software used in Rodriguez et al. (2021), which is the SOP we used for this study. However, the process of tracing patches of coral tissue in ArcGIS Pro is entirely manual, which necessitates many person-hours for tracing. To expedite our workflow, a subset of researchers explored whether the emerging freeware platform TagLab [8] could reduce tracing time without compromising accuracy.

TagLab is a semantic segmentation platform that allows users to delineate the boundaries of benthic organisms and assign species ID. TagLab’s “positive-negative click” tool assists the annotator by providing a “best guess” of colony boundaries based on RITM segmentation [9]. To do this, the annotator selects several pixels within (+) and outside (-) of a patch of live coral tissue. Each click modifies the “best guess” tracing that TagLab generates, which can be modified with successive positive or negative clicks until the annotator is satisfied with the delineation. In our study, we used the positive-negative click tool as a starting point for tracing, and manually edited coral boundaries as needed.

While seven different annotators traced corals for this project, all annotations were edited and quality checked by a single annotator (OM) for consistency within TagLab. To test for potential differences in accuracy caused by software or annotator identity, we instructed one annotator from Scripps Institution of Oceanography (SIO) and Arizona State University (ASU) to trace all *Pocillopora* colonies at one site (Molokini). Comparing traced boundaries of the same corals, we found no meaningful difference in planar area between the SIO and ASU annotator (Figure S3), which were using their organization’s respective software of choice (TagLab for SIO, ArcGIS Pro for ASU).

**References**

1. Liu G, Skirving WJ, Geiger EF, De La Cour JL, Marsh BL, Heron SF, et al. NOAA Coral Reef Watch’s 5km Satellite Coral Bleaching Heat Stress Monitoring Product Suite Version 3 and Four-Month Outlook Version 4. Reef Encount. 2017;32: 39–45. Available: https://coralreefwatch.noaa.gov/satellite/bleachingoutlook_cfs/outlook_cfs.php.

2. Skirving W, Marsh B, De La Cour J, Liu G, Harris A, Maturi E, et al. Coraltemp and the coral reef watch coral bleaching heat stress product suite version 3.1. Remote Sens. 2020;12: 1–10. doi:10.3390/rs12233856

3. Pandolfi JM, Connolly SR, Marshall DJ, Cohen AL. Projecting coral reef futures under global warming and ocean acidification. Science (80- ). 2011;333: 418–422. doi:10.1126/science.1204794

4. Kayanne H. Validation of degree heating weeks as a coral bleaching index in the northwestern Pacific. Coral Reefs. 2017;36: 63–70. doi:10.1007/s00338-016-1524-y

5. Couch CS, Burns JHR, Liu G, Steward K, Gutlay TN, Kenyon J, et al. Mass coral bleaching due to unprecedented marine heatwave in Papahānaumokuākea Marine National Monument (Northwestern Hawaiian Islands). PLoS One. 2017;12. doi:10.1371/journal.pone.0185121

6. Powell B. Regional Ocean Modeling System (ROMS): Main Hawaiian Islands. April 15, 2015 to December 31, 2021. Distributed by the Pacific Islands Ocean Observing System (PacIOOS). 2010. Available: http://pacioos.org/metadata/roms_hiig.html.

7. Rodriguez C, Amir C, Gray A, Asbury M, Suka R, Lamirand M, et al. Measuring Coral Vital Rates Using Photogrammetry at Fixed Sites : Standard Operating Procedures and Error Estimates Measuring Coral Vital Rates Using Structure-from-Motion Photogrammetry at Fixed Sites : Standard Operating Procedures and Error Estimates. 2021.

8. Pavoni G, Corsini M, Ponchio F, Muntoni A, Edwards CB, Pedersen NE, et al. TagLab: AI-assisted annotation for the fast and accurate semantic segmentation of coral reef orthoimages. J F Robot. 2021. doi:10.1002/rob.22049

9. Sofiiuk K, Petrov IA, Konushin A. Reviving Iterative Training With Mask Guidance for Interactive Segmentation. Proc - Int Conf Image Process ICIP. 2022;1071: 3141–3145. doi:10.1109/ICIP46576.2022.9897365


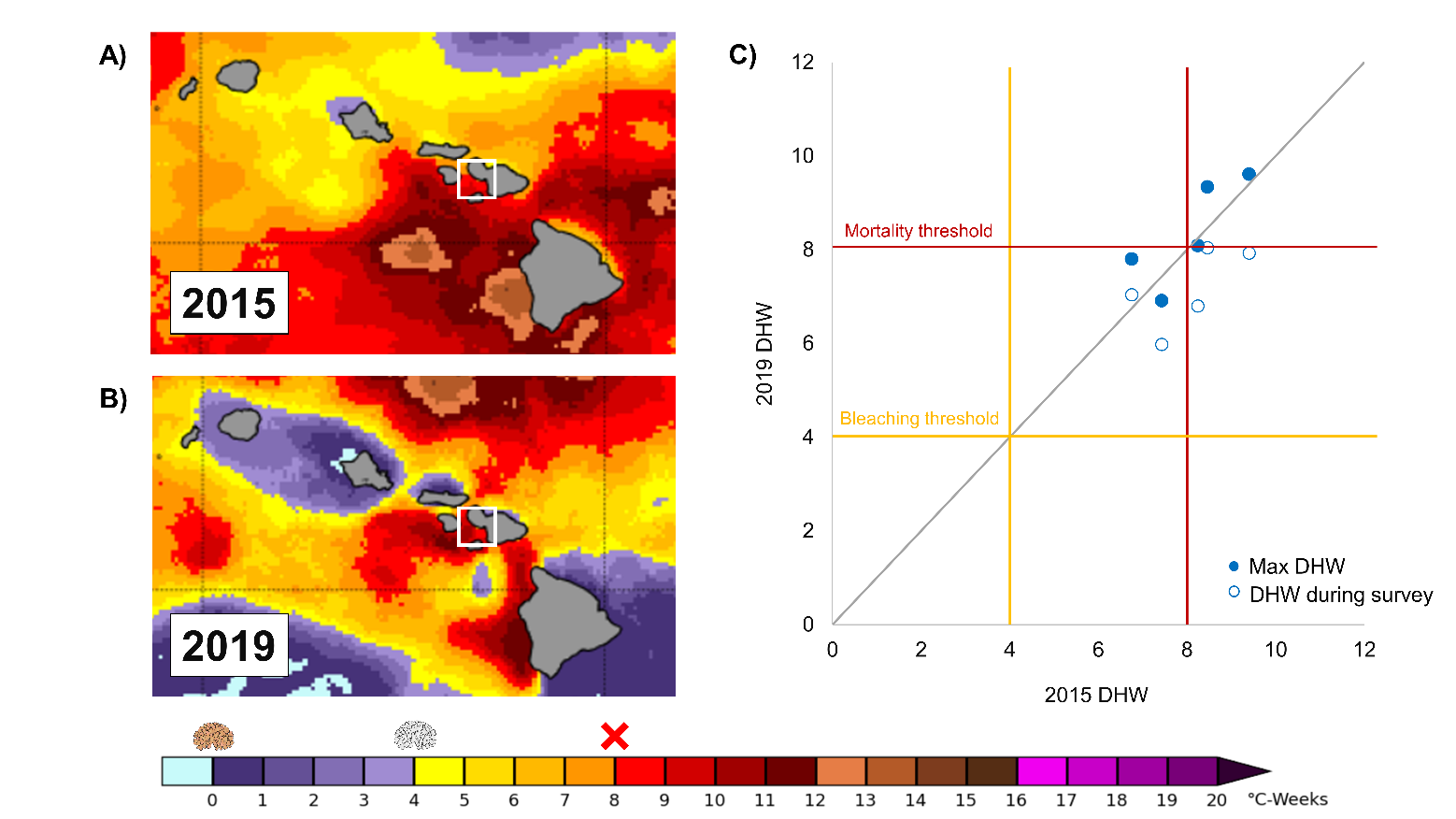


**S1 Fig.** The maximum degree heating weeks (DHW) at study sites is shown for both 2015 and 2019. Points that fall on the gray diagonal line in experienced the same DHW in both 2015 and 2019. Expected thresholds for coral bleaching and mortality are shown in yellow and red respectively. Thermal stress was comparable in leeward Maui among sites and between bleaching events. All sites surpassed 4 DHW in 2015 and 2019, indicating that widespread bleaching was expected in both years. Kahekili, Wahikuli, and Ukumehame did not reach the 8 DHW threshold for mortality in 2015 or 2019, while Olowalu, Keawakapu, and Molokini surpassed that threshold in both 2015 and 2019.


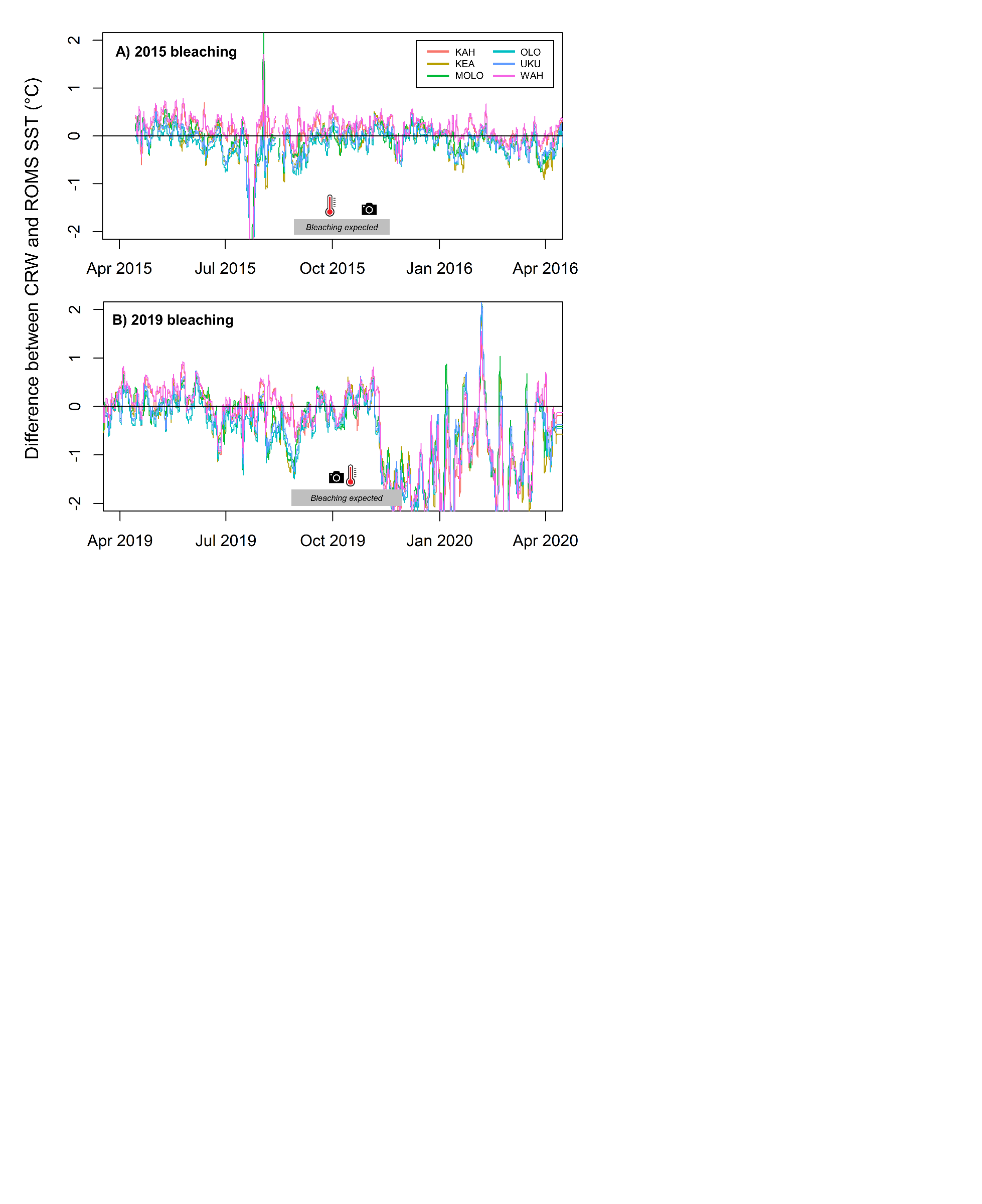


**S2 Fig**. A comparison of ocean temperature from Coral Reef Watch (CRW) and the Regional Ocean Modelling System (ROMS) for the duration of the bleaching events in (A) 2015 and (B) 2019. Temperature timeseries are color coded by site. Positive values indicate that ROMS forecasts were warmer than CRW temperature values, while negative values indicate cooler ROMS forecasts compared to CRW. CRW sea surface temperature is derived from satellites, while ROMS incorporates both satellite and *in situ* data sources into its forecasts. The gray bar indicates the range of dates with > 4 degree heating weeks (DHW), which indicates that bleaching would be expected. The thermometer symbol indicates the date where degree heating weeks peaked, after which DHW plateaued through October before falling in November (same pattern and timing in both years). The camera symbol indicates the date(s) where we resurveyed our long-term monitoring sites.


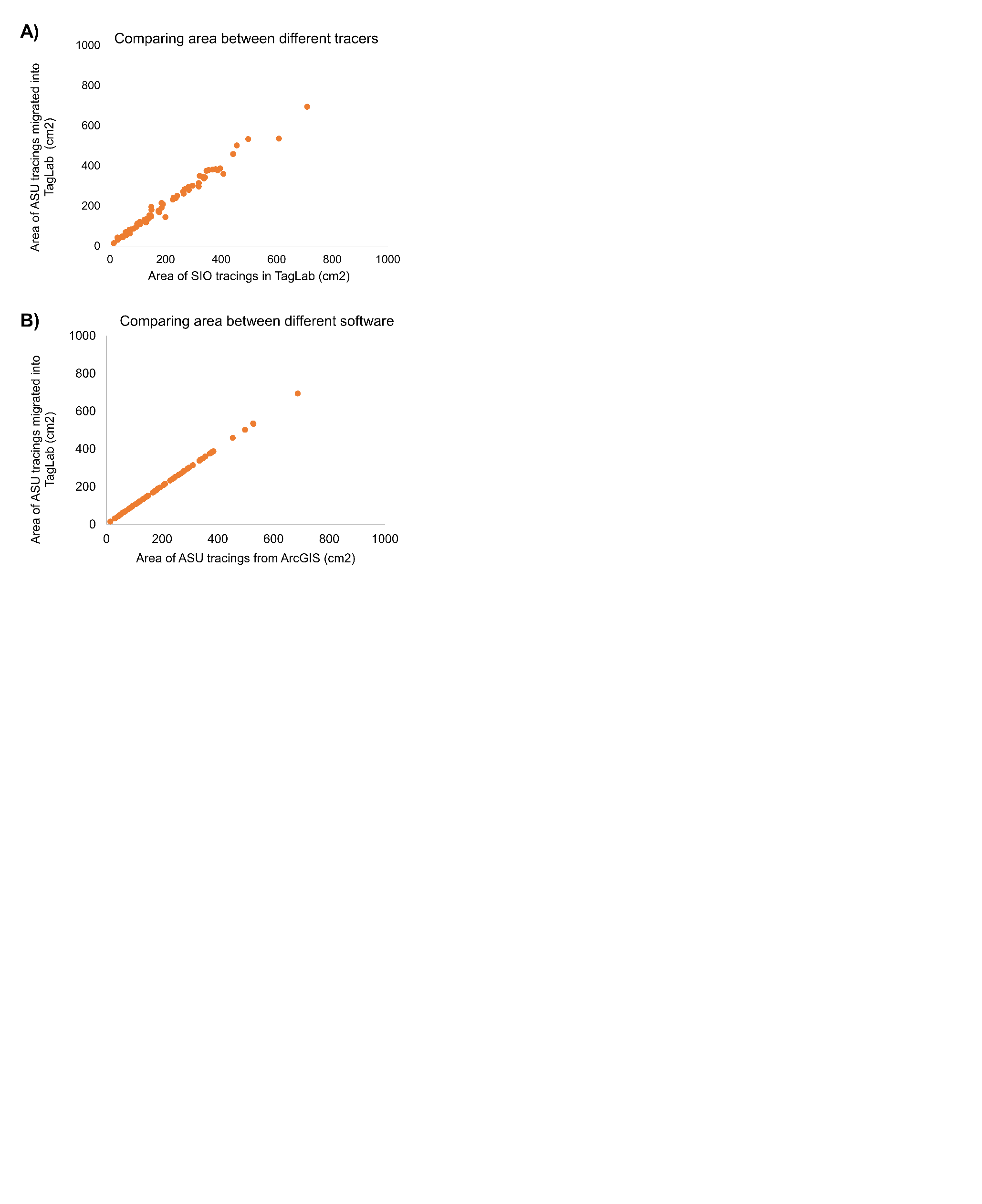


**S3 Fig.** *Pocillopora* colony planar area compared (A) between different annotators within TagLab, and (B) between the same annotator within ArcGIS Pro and TagLab. SIO stands for Scripps Institution of Oceanography, and ASU stands for Arizona State University.


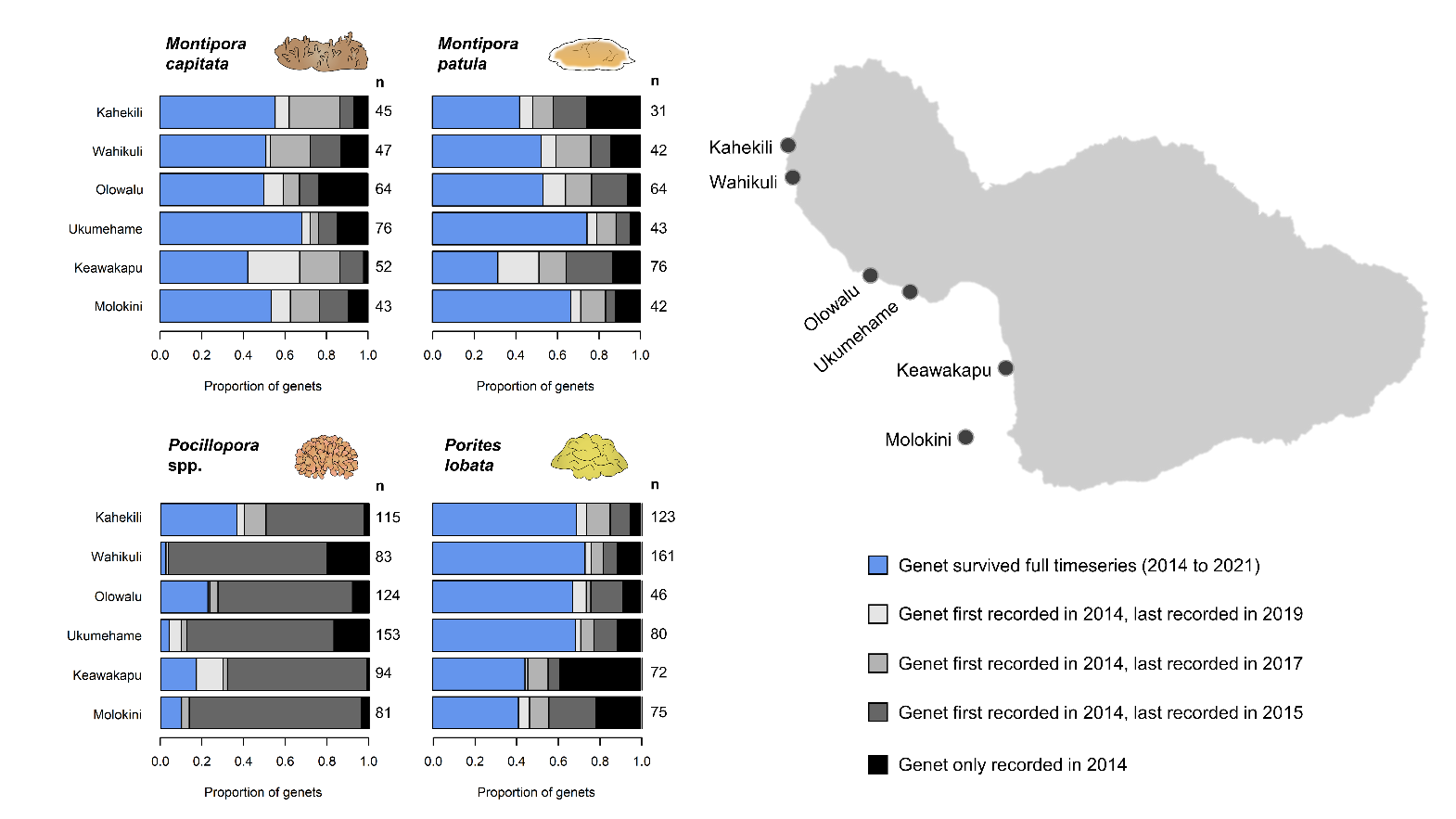


**S4 Fig.** All genets are shown for each coral population, with blue representing the proportion of all genets that survived the full duration of the timeseries (2014 to 2021). Genets that did not survive are denoted with shades of grey indicating the last year they were recorded in the timeseries.


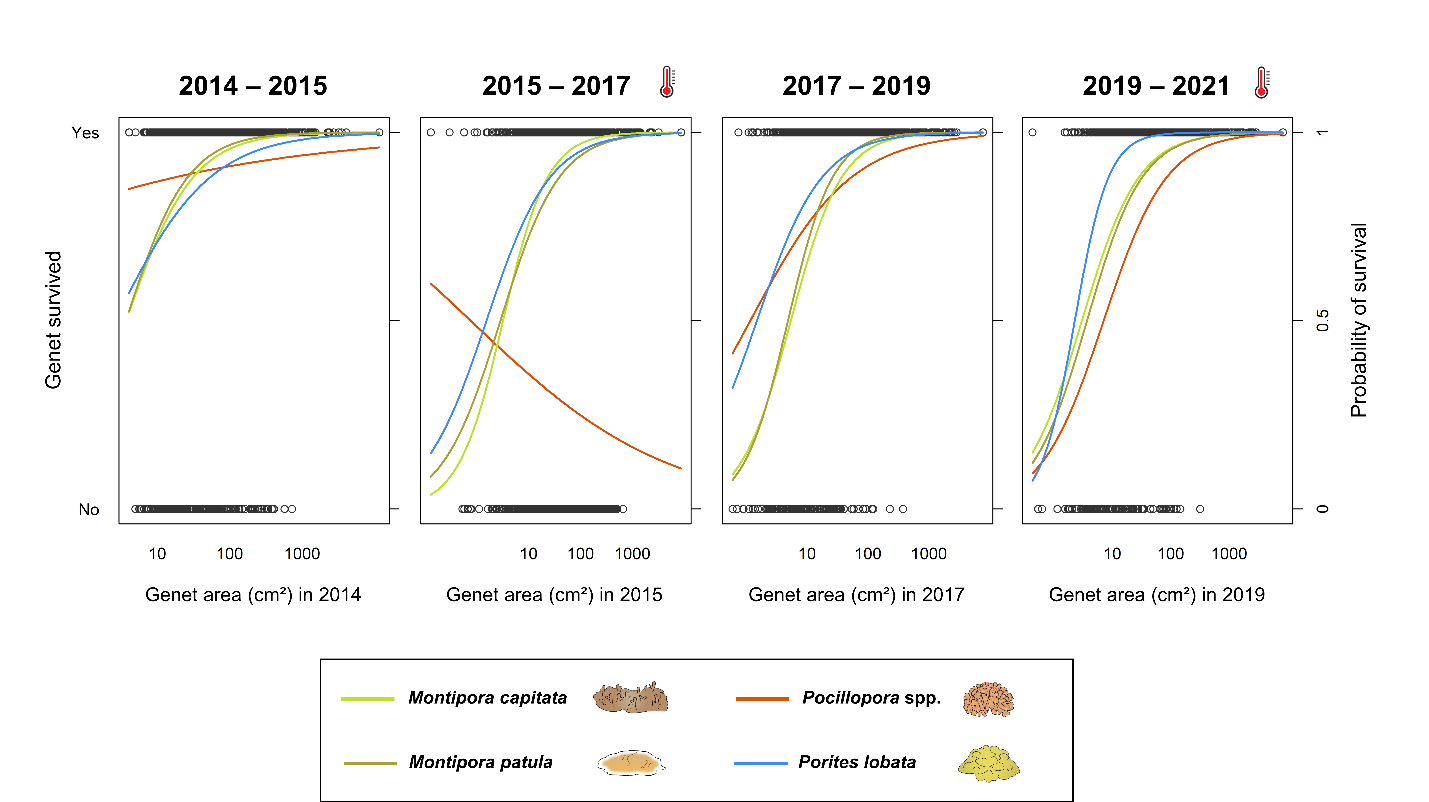


**S5 Fig.** The relationship between genet planar area and survivorship is shown for each timestep. Colors denote coral taxa and thermometer icons denote timesteps following documented bleaching events. Survivorship was highest during the first timestep for all taxa, and declined following bleaching in 2015. The relationship between planar area and survivorship for *Pocillopora* inverted multiple times, while for other taxa it remained positive over the entire timeseries.

**S1 Table.** Metadata for each study site, including coordinates, depth, distance from shore, coral cover at the start and end of the timeseries, and degree heating weeks (DHW) during 2015 and 2019 (both during our large-area imaging surveys and at the peak of the bleaching event).

| Site | Lat. | Long. | Depth (m) | Dist. from shore (m) | 2014 coral cover (%) | 2021 coral cover (%) | Max DHW (2015) | DHW during survey (2015) | Max DHW (2019) | DHW during survey (2019) |
| --- | --- | --- | --- | --- | --- | --- | --- | --- | --- | --- |
| Kahekili | 20.9368 | -156.6937 | 5.2 | 74 | 62.56 | 54.26 | 6.75 | 6.75 | 7.8 | 7.04 |
| Keawakapu | 20.7038 | -156.4504 | 10.0 | 341 | 52.99 | 34.34 | 8.24 | 8.24 | 8.09 | 6.8 |
| Molokini | 20.6315 | -156.4966 | 8.7 | 41 | 82.09 | 71.64 | 9.4 | 9.4 | 9.61 | 7.93 |
| Olowalu | 20.8047 | -156.6072 | 9.7 | 487 | 52.55 | 43.51 | 8.46 | 8.46 | 9.34 | 8.04 |
| Ukumehame | 20.7910 | -156.5843 | 8.6 | 460 | 43.72 | 44.03 | 7.43 | 7.43 | 6.91 | 5.98 |
| Wahikuli | 20.9098 | -156.6917 | 4.5 | 174 | 40.22 | 40.30 | 6.75 | 6.75 | 7.8 | 7.04 |

**S2 Table.** A frequency table showing the sample size of genets for each taxon and site, as well as the number and proportion of those genets that survived until the end of the timeseries (2021). The number of 0.5 m^2^ quadrats and the associated survey area is also noted for each population. We did not use quadrats to survey *Pocillopora* (given the low density of this genus on the reef) and instead traced every *Pocillopora* within each 12x12 m orthoprojection. Total survey area for *Pocillopora* is lower than 144 m^2^ for some populations due to gaps in the orthoprojection in some years. For consistency, when part of an orthoprojection was missing in a given year, we did not trace colonies from that section of reef in other years.

|  | Site | Genets tracked (n) | Survivors (n) | Proportion surviving | Quadrats  (n) | Survey area (m^2^) |
| --- | --- | --- | --- | --- | --- | --- |
| *Montipora capitata* | Kahekili | 45 | 25 | 0.56 | 10 | 5 |
|  | Keawakapu | 52 | 22 | 0.42 | 13 | 6.5 |
|  | Molokini | 43 | 23 | 0.53 | 11 | 5.5 |
|  | Olowalu | 64 | 32 | 0.50 | 10 | 5 |
|  | Ukumehame | 76 | 52 | 0.68 | 10 | 5 |
|  | Wahikuli | 47 | 24 | 0.51 | 11 | 5.5 |
| *Montipora patula* | Kahekili | 31 | 13 | 0.42 | 25 | 12.5 |
|  | Keawakapu | 76 | 24 | 0.32 | 13 | 6.5 |
|  | Molokini | 42 | 28 | 0.67 | 11 | 5.5 |
|  | Olowalu | 64 | 34 | 0.53 | 10 | 5 |
|  | Ukumehame | 43 | 32 | 0.74 | 10 | 5 |
|  | Wahikuli | 42 | 22 | 0.52 | 20 | 10 |
| *Pocillopora* spp. | Kahekili | 115 | 42 | 0.37 | - | 144 |
|  | Keawakapu | 94 | 16 | 0.17 | - | 139 |
|  | Molokini | 81 | 8 | 0.10 | - | 125 |
|  | Olowalu | 124 | 28 | 0.23 | - | 143 |
|  | Ukumehame | 153 | 6 | 0.04 | - | 127 |
|  | Wahikuli | 83 | 2 | 0.02 | - | 137 |
| *Porites lobata* | Kahekili | 123 | 85 | 0.69 | 10 | 5 |
|  | Keawakapu | 72 | 32 | 0.44 | 17 | 8.5 |
|  | Molokini | 75 | 31 | 0.41 | 19 | 9.5 |
|  | Olowalu | 46 | 31 | 0.67 | 23 | 11.5 |
|  | Ukumehame | 80 | 55 | 0.69 | 10 | 5 |
|  | Wahikuli | 161 | 118 | 0.73 | 10 | 5 |

**S3 Table.** Pairwise comparisons of genet survivorship between sites over the timeseries (2014 to 2021). Comparisons are only shown for site pairs that did not exhibit an interaction. P values have been adjusted for multiple comparisons using the Bonferroni method.

|  | Contrast | Estimate | SE . | P value | Sig |
| --- | --- | --- | --- | --- | --- |
| *Montipora capitata* | Kahekili - Keawakapu | 0.964 | 0.473 | 0.622 | n.s. |
|  | Kahekili - Molokini | 0.982 | 0.548 | > 0.999 | n.s. |
|  | Keawakapu - Ukumehame | -2.353 | 0.554 | **< 0.001** | ******* |
|  | Keawakapu - Wahikuli | -0.858 | 0.477 | > 0.999 | n.s. |
|  | Molokini - Olowalu 3 | -0.666 | 0.527 | > 0.999 | n.s. |
|  | Molokini - Ukumehame | -2.371 | 0.620 | **0.002** | ****** |
|  | Olowalu 3 - Ukumehame | -1.706 | 0.549 | **0.028** | ***** |
| *Montipora patula* | Kahekili - Keawakapu | 0.631 | 0.526 | > 0.999 | n.s. |
|  | Kahekili - Ukumehame | -1.200 | 0.600 | 0.681 | n.s. |
|  | Kahekili - Wahikuli | -0.432 | 0.591 | > 0.999 | n.s. |
|  | Keawakapu - Molokini | -0.878 | 0.493 | > 0.999 | n.s. |
|  | Keawakapu - Ukumehame | -1.831 | 0.471 | **0.002** | ****** |
|  | Keawakapu - Wahikuli | -1.063 | 0.460 | 0.312 | n.s. |
|  | Molokini - Olowalu 3 | 0.514 | 0.518 | > 0.999 | n.s. |
|  | Ukumehame - Wahikuli | 0.768 | 0.543 | > 0.999 | n.s. |
| *Pocillopora* spp. | Kahekili - Keawakapu | 1.204 | 0.398 | **0.037** | ***** |
|  | Kahekili - Olowalu 3 | 0.626 | 0.309 | 0.637 | n.s. |
|  | Kahekili - Ukumehame | 2.602 | 0.475 | **< 0.001** | ******* |
|  | Keawakapu - Olowalu 3 | -0.578 | 0.396 | > 0.999 | n.s. |
|  | Keawakapu - Ukumehame | 1.397 | 0.536 | 0.138 | n.s. |
|  | Molokini - Wahikuli | 1.587 | 1.135 | > 0.999 | n.s. |
|  | Olowalu 3 - Ukumehame | 1.975 | 0.474 | **< 0.001** | ******* |
| *Porites lobata* | Kahekili - Keawakapu | 1.468 | 0.438 | **0.012** | ***** |
|  | Kahekili - Molokini | 2.329 | 0.453 | **< 0.001** | ******* |
|  | Keawakapu - Molokini | 0.862 | 0.443 | 0.778 | n.s. |
|  | Keawakapu - Ukumehame | -1.282 | 0.446 | 0.061 | n.s. |
|  | Keawakapu - Wahikuli | -1.602 | 0.437 | **0.004** | ****** |
|  | Molokini - Ukumehame | -2.144 | 0.461 | **< 0.001** | ******* |
|  | Molokini - Wahikuli | -2.464 | 0.452 | **< 0.001** | ******* |

**S4 Table.** A summary of the best and worst performing coral populations with respect to their bleaching response over time, growth rates, and probability of survivorship. Coral populations with significantly higher proportions of thermally tolerant or acclimatized genets, significantly higher rates of growth, and significantly higher probabilities of survivorship are categorized here as “most resilient”. Kahekili and Olowalu has the most evidence of resilient coral populations, while Molokini and Keawakapu has the least.

|  | Taxa | Bleaching | Growth | Survivorship |
| --- | --- | --- | --- | --- |
| *Best performing sites* | *Montipora capitata* | Kahekili | Molokini (small genets) | Ukumehame |
|  | *Montipora patula* | Olowalu | Kahekili  Wahikuli  Molokini  Olowalu | Ukumehame |
|  | *Pocillopora* spp. | Kahekili  Olowalu  Keawakapu | Olowalu | Kahekili |
|  | *Porites lobata* | Olowalu | Kahekili  Wahikuli  Olowalu  Ukumehame | Kahekili  Ukumehame  Wahikuli |
| *Worst performing sites* | *Montipora capitata* | Olowalu  Ukumehame  Wahikuli  Molokini | Keawakapu (large genets) | Olowalu  Molokini  Keawakapu |
|  | *Montipora patula* | Ukumehame  Wahikuli | Keawakapu | Keawakapu |
|  | *Pocillopora* spp. | Not enough survivorship to assess bleaching responses at Wahikuli, Ukumehame, and Molokini | Keawakapu | Ukumehame  Molokini  Wahikuli |
|  | *Porites lobata* | Wahikuli | Molokini | Keawakapu  Molokini |
