# Supplementary material for: Corals that survive repeated thermal stress show signs of selection and acclimatization
Source: PLoS One. 2024 Jul 31;19(7):e0303779. doi: 10.1371/journal.pone.0303779 (PMC11290665; doi:10.1371/journal.pone.0303779)
Supplement: Attachment — Submitted filename: 2024.04 Response to reviewers.docx [file pone.0303779.s002.docx]

**Reviewer 1**

This study focusses on bleaching responses of nearly 2000 colonies of four coral species over seven years, overlapping two mass bleaching events in 2015 and 2019 at Maui. By examining bleaching extent, growth and survivorship of each coral through large-area photogrammetry, the authors found limited support for acclimatisation of populations within and among reefs. Individual colonies may be more tolerant and have lower bleaching extents over time, but there was generally no relationship between bleaching and coral growth.

The approach to track individual colonies is appropriate for testing acclimatisation at various scales—from colony to reef—and the application of photogrammetry over large areas does generate a much larger amount of data relative to conventional reef surveys. Taxonomic resolution may be limited, but it is sufficient for tracking four easily recognisable taxa. The text is well written and figures generally clear and support the results well, despite the poor resolution, likely a limitation of the manuscript submission system. I have a few comments and minor suggestions that hopefully will help clarify and improve the manuscript.

The coral colonies are holobionts that comprise the host, Symbiodiniaceae endosymbionts and microbial communities. While the characterisation of these components is understandably beyond the scope of this study, their contributions to thermal tolerance and resilience are significant and should be discussed.

This is a good suggestion. We restructured lines 84-90 in response to your other comment about describing specific studies of acclimatization. As part of that paragraph, we added text about the contribution of microbial communities to coral acclimatization potential:

“In addition, the microbial communities associated with a coral “holobiont” can perform beneficial functions, such as nitrogen fixation and reactive oxygen scavenging, that have been shown to improve coral survivorship during heat stress (Santono et al. 2021).”

The distinction between the effects of coral acclimatisation or taxonomic turnover on thermal tolerance at the reef level is also alluded to at various parts of the manuscript, but this does not appear to be tested specifically. Could the abundance changes between surveys be modelled to test for temporal variations in relative abundances of taxa and the effects on subsequent bleaching?

Thanks for this question! In short, we saw evidence for both acclimatization and taxonomic turnover. Given that the occurrence of acclimatization doesn’t preclude taxonomic turnover from occurring (and visa versa), it feels strange to us to test whether one process is occurring over the other (they are both occurring). It’s also worth noting that our data do not reflect the relative abundance of taxa on the reef, since our surveys were designed to capture a roughly equal sample size for each taxon, despite large differences in the relative abundance of these taxa (each reef had dozens of Pocillopora genets but thousands of Porites genets, for example).

Furthermore, to address your suggestion to test for temporal variations in relative abundances of taxa and the effects on subsequent bleaching, we kindly refer you to figures S4 and S5 (previously S5 and S6 in our first submission). These figures show how heat stress caused disproportionate mortality of certain taxa (i.e., Pocillopora) and size classes (small genets, except for Pocillopora where large genets were more impacted). Impacts from the 2015 bleaching event shaped population demographics going forward, and this can be seen in the inversion of the Pocillopora size-mortality relationship during the 2019 bleaching event.

The inability to distinguish the Pocillopora spp. is slightly challenging for the inferences associated with population-level changes, primarily because these are masked by the corals being lumped as one taxonomic group. This limitation should be explicitly factored into the uncertainties surrounding results on Pocillopora.

This is a good point. We have added the following sentence to the discussion to indicate this source of uncertainty:

“While Pocillopora survivors showed patterns of reduced bleaching consistent with acclimatization at certain sites, we are unable to say what mechanism is responsible for those shifts. It is possible that cryptic species diversity could explain site-level differences in bleaching, given that we were only able to identify *Pocillopora* genets to the genus level.”

A couple of minor issues on nomenclature: it should be scleractinian (word not capitalised) and Scleractinia (formal name with word capitalised); and do not capitalise family names, e.g. Symbiodiniaceae.

Text changed accordingly.

Abstract
This is well-written, but I thought the implications for restoration are not represented here. For example, the point that targeting specific phenotypes of Montipora or Porites based on thermal resistance may not lead to better outcomes should be mentioned here. Overall, the implications are written slightly too generally to be informative.

Thank you for this suggestion. We have added the following sentence to the abstract to more specifically call out the implications of our results for restoration:

“Based on these results, we recommend restoration practitioners in Hawaiʻi focus on colonies of *Porites* and *Montipora* with a proven track-record of growth and survivorship, rather than devote resources toward identifying and cultivating bleaching-resistant phenotypes in the lab.”

Lines 84–90
Rather than listing mechanisms and areas of study, it would be helpful to describe a couple of studies and their results as examples for understanding acclimatisation in corals.

Thank you for this suggestion. We have removed the list of acclimatization mechanisms and restructured the paragraph to read as follows:

“Acclimatization refers to the process of phenotypic or epigenetic change that occurs within the lifespan of a single individual to cope with environmental stress [36]. In corals, acclimatization to heat stress can occur through a variety of mechanisms. For instance, Million et al. (2022) tracked *Acropora cervicornis* outplants through a bleaching event in Florida and found that genotypes with higher morphological plasticity had lower mortality and higher growth rates. Furthermore, these relationships strengthened over the course of the experiment, suggesting that acclimatization was actively occurring in response to heat stress [37]. In Kiribati, Claar et al. (2020) observed colonies of *Platygyra ryukyuensis* and *Favites pentagona* switch their symbiont communities from *Cladocopium* to *Durisdinium* during a bleaching event. This symbiont shuffling allowed colonies to regain pigmentation and recover from bleaching while heat stress remained elevated [38]. Heat-responsive genes have been found in coral tissues as well, and there is evidence that corals can modulate expression of these genes in response to heat stress [39,40]. In addition, the microbial communities associated with a coral “holobiont” can perform beneficial functions, such as nitrogen fixation and reactive oxygen scavenging, that have been shown to improve coral survivorship during heat stress [41].”

Lines 127–130
Again here, a description of results of these studies would be constructive. In particular, why ‘but see Fukunaga et al. 2022’?

We cited Fukunaga et al. 2022 here because it was the only study we could find that also documents sequential bleaching events, tracks individual corals using large-area imagery, and quantifies bleaching/growth/survivorship in demographically complex taxa. Thus, the results of Fukunaga et al. 2022 are less relevant here; rather we want to emphasize Fukunaga’s methods and overarching study design.

Furthermore, earlier it has been asked if changes in thermal tolerance could be driven by coral acclimatisation or taxonomic turnover. Could this be addressed with the analysis here?

See response to earlier comment about acclimatization and taxonomic turnover.

Line 148
It would be clearer to have a temperature graph for each of the 5 years of surveys, indicating the survey date—something like Figure S2 but for all survey years, to have an understanding of the severity of the heat stress. And this should be in the main text.

Thank you for this suggestion. We created a series of plots showing the SST anomaly and DHW across our six sites for each year. To simultaneously address your suggestion and another suggestion from reviewer 2 to elevate Figure S4 into the main manuscript, we merged these thermal stress plots with the population bleaching plots in Figure S4, and have moved this figure to the main text (now figure 1, and other figure names have been adjusted as a result). We feel that this new composite figure provides a good overview of heat stress and population bleaching responses overall, making it a good starting point for discussions around acclimatization and selection. The new caption for this figure is as follows:

**Fig. 1. Bleaching response of coral populations in each survey year.** Thermal stress is shown in each survey year for all sites in terms of (A) SST anomaly with respect to the 1985-2012 climatological mean, and (B) degree heating weeks (DHW). The black dotted line in (A) indicates a SST anomaly of 0. The yellow and red dotted lines in (B) denote the DHW threshold where bleaching and mortality, respectively, are expected to occur. Camera icons in (A-B) indicate the timing of large-area imagery surveys. (C) The bleaching response of each coral population is shown in each year. All genets are included, not just those that survived to the end of the timeseries. Thermometer icons denote years with documented bleaching events.

Line 191
It’s not clear the necessary to mention ‘Researchers from both Scripps Institution of Oceanography (SIO) and Arizona State University (ASU) used the orthoprojections to delineate patches of live coral tissue.’

Text removed.

Line 210
Is this use of ‘patches’ problematic for defining genets when you have partially dead corals with patchy live tissue left on the original colony? Would separate ‘patches’ from the same colony (previously) be considered different genets, and does this become a pseudoreplication problem?

You bring up a good point. Identifying genets based on the fission/fusion dynamics of patches could lead to pseudoreplication in cases where a formerly large colony split into multiple small patches prior to our timeseries if those patches never re-fuse. During our initial analyses, we looked through the traced patches and resulting genet data to see if we could identify obvious instances of the pseudoreplication you describe. We found very few, likely for three reasons. First, we use a size threshold of 5 cm in diameter in 2014 as a criteria for including coral patches in our study. Thus, smaller patches that resulted from fragmentation prior to 2014 would not be included here. Second, our first study year (2014) predates the main driver of partial mortality and fragmentation (the 2015 bleaching event), thus our timeseries captures the “starting point” of most colonies, with most in a pre-fragmented state. Third, our study describes patterns of acclimatization using only genets that survived the full timeseries. Given that smaller corals were less likely to survive the full duration of the timeseries than larger ones, those smaller patches that failed to refuse with other patches largely died out before the end of the timeseries, reducing instances of the pseudoreplication you describe. Finally, while it is true psuedoreplication could be an issue here, the approach based on fission/fusion dynamics is currently the best available option based on the imagery we have available, short of genetic testing (which was outside the scope and funding of this study), and has been used in other studies (Brito-Millan et al. 2019, <https://www.jstor.org/stable/26920540?seq=4>).

To address this valid concern, we have added the following sentence after line 210: “Additionally, we visually inspected orthoprojections to identify potential instances of pseudoreplication arising from colony fission prior to our timeseries, and found minimal evidence to support this concern.”

Line 232
‘Focusing on this bleached tissue only, we then scored bleaching severity (from 0 to 3) based on the overall degree of paling observed (0 for < 5% of pigmentation lost; 1 for 5 – 50%, 2 for 50 – 95%, and 3 for > 95%; Rodriguez et al. 2021).’
Is this measure based on colouration? What colour parameters are being used? E.g. what does 10% or 50% of paling mean? The current description is rather vague.

Thank you for pointing that the use of percentages is confusing here. We have removed the percentages and instead use the severity categories as written in Rodriguez et al: “Focusing on this bleached tissue only, we then scored bleaching severity (from 0 to 3) based on the overall degree of paling observed (0 for practically no pigmentation loss; 1 for slight paling, 2 for significant loss of pigmentation, and 3 for almost or completely stark white; Rodriguez et al. 2021).”

Line 319
forin?

Thank you for catching that typo. Change to “in”.

**Reviewer 2**

The study performed by McCarthy et al., in my opinion is very interesting and well structured, as well as original and innovative in terms of methodology. All the technical and experimental procedures seem to have been performed in a precise and reliable way and the results showed are considerable in term of both quantity and quality. The work proposes some interesting findings that surely merit publication, also considering the constant increase in the intensity and frequency of bleaching events worldwide and the future challenges that corals will face in the context of global climate change. In addition, in my opinion, the main strong point of the work is the long monitoring period and the high number of colonies analysed. The paper is also well written. For all these reason, I suggest to the Editor to accept the manuscript following a minor revision.

However, the main problem with this article is its length. Authors should cut some text in all the manuscript paragraphs and make the manuscript easier and more fluid to read. Many information, analysis and results are included in the manuscript, and the authors should try to make them more easily digestible for the reader.

Here, some specific comments section by section.

Introduction:
1) Pag. 3, Lines 64-65. “Still, scleractinian….(Pandolfi et al 2011). I would delete this sentence

Done.

2) Pag. 3, Lines 65-68. “More recently….still a possibility”. I would suggest to move this sentence after the line 79 (Pag. 4).

Thank you for the suggestion. We would like to keep this sentence where it is since it focuses on coral resilience to generalized stressors, not specifically to bleaching (whereas paragraph 2 is focused specifically on bleaching). It is also helpful for ending paragraph 1 on a positive and hopeful note, which is appropriate given that this study documents hopeful signs of acclimatization and population persistence through bleaching.

3) Pag. 4, Line 87. I would add as mechanism for acclimatization to heat stress also the coral physiological and molecular features (see for example Louis et al., 2020 Molecular Ecology, Bellantuono et al., 2012 PlosONE, Traylor-Knowles et al., 2017 JEB….).

Thanks for suggesting these references. We restructured this paragraph somewhat (see response to reviewer 1’s comment about lines 84-90) and have included references to Bellantuono et al. 2012 and Traylor-Knowles et al. 2017.

“… Beyond symbionts, heat-responsive genes have been found in coral tissues as well, and there is evidence that corals can modulate expression of these genes in response to heat stress (Bellantuono et al. 2012, Traylor-Knowles et al. 2017).”

4) Pag. 4 , Line 91. I would suggest to add also Maldives in the list (see Seveso et al. 2018, Coral Reefs)

Done.

5) Pag. 4, Line 96. I would delete the ref. Winston et al in prep throughout all the manuscript

Winston et al. in prep is a companion study to this work. Both studies are a result of a collaborative research effort between Scripps and Arizona State University to track the response of corals in Hawaii to bleaching. While this study focuses on the impact of both the 2015 and 2019 bleaching events in Maui specifically, Winston et al. uses data from multiple Hawaiian Islands to study the short-term impact of the 2019 bleaching event on coral survivorship, and it is currently in the final stages of submission for publication in Marine Ecology Progress Series. Thus, Winston et al. brings a broader spatial, if more limited temporal, perspective and looks into different but related questions related to the ecological and environmental predictors of survivorship. Many of its findings are highly relevant to the results presented here, and as a companion piece of research, it feels informative and transparent to direct readers to it, especially in instances where it can shed additional light on observations that we only briefly mention here.

6) Pag. 6, Lines 132-134. Since they are questions, why is the question mark missing?

Reworded this compound question, splitting it into three separate sentences: “Specifically, we sought to answer three questions. First, do individual corals, as well as coral populations, show evidence of acclimatization? Second, do corals with lower rates of bleaching outperform other corals over time? Finally, do different coral populations show similar responses to sequential heat stress in terms of bleaching, growth, and survivorship?”

Methods:
1) I would insert as figure a map of the study area in which also the different analyzed sites are indicated.

Thank you for this suggestion. A map of our study sites is already included as part of Figure 6. Given that we have seven figures in the main manuscript (with the elevation of Figure S4) we don’t feel that it is appropriate to add an additional figure that duplicates the map in Figure 6.

2) Pag. 7, Lines 167-168. I would add as Reference also Montalbetti et al 2022, Coral Reefs

The other references (D’Urban Jackson et al. 2020; Bongaerts et al. 2021; Ferrari et al. 2021; McCarthy et al. 2023) are all review papers that broadly discuss how large-area imagery has been applied to study coral reef to date. We feel that these studies are appropriate to support our generalized claim about large-area imagery applications, more so than case studies that apply large-area imagery to study a specific process in a specific place (like Montalbetti et al. 2022).

3) Pag. 7, Lines 170-189. This part is too long. Please, shorten it.

We streamlined lines 170-180 to read: “At each fixed site, divers entered the water with two D7000 SLR Nikon cameras (focal lengths of 18mm and 55mm) in Ikelite underwater housings. Divers marked the boundaries of each 10 x 10 m long term monitoring plot with six calibration tiles, and placed four 0.5 m long scale bars within the plot. One diver swam approximately 1.5 m over the reef in a gridded pattern with the cameras, which were programmed to take a picture every second. This produced approximately 5,000 pictures per site. The camera diver imaged a core area of 10x10 m, and swam several meters beyond the calibration tiles to ensure that a buffer region (> 1 m wide) around the core area was also imaged.”

The information included in lines 181-189 is all critical to our methods and has already been heavily abridged in terms of detail, so we elected to leave that text as is.

4) Pag. 8, Lines 191-196. Is this information really necessary? If yes, try to write it differently

Lines 191-196 were streamlined to read “Researchers used TagLab and ArcGIS Pro to trace patches of live coral tissue following the approach of Rodrguez et al. 2021. We found no effect of software on traced planar area (Figure S3), and a single annotator (OM) QCed all tracings in TagLab to control for any potential effect of annotator or software.”

5) Pag. 10, Lines 244-247. I would delete the part in brackets.

Done.

Results:
In general, try to shorten all the result paragraphs (especially those related to Growth and Survivorship)

Thank you for this suggestion. We have removed text in several places, including from the paragraph on growth and survivorship. Omissions are clearly noted in the attached track changes document.

1) I would move the Fig. S4 in the Figure list of the manuscript

Done. This is now Figure 1. Also, see response to reviewer 1 re: line 148.

2) Pag. 13, Line 325. “…although the small sample size.”

Reworded the sentence to read “*Pocillopora* that survived until 2019 from Ukumehame demonstrated increasing bleaching susceptibility over time, although the sample size for that population was small.”

3) Pag. 13, Lines 326-327. “Pocillopora…..event in 2015”. I would move this sentence to the Legend of Figure 4

Done.

4) Pag. 16, Line 394. Delete this sentence

Sentence deleted. Modified the following sentence to read “We identified sites with the highest and lowest survivorship through pairwise comparisons of sites that did not exhibit significant interactions.”

Discussion:
The discussion is very exhaustive and well written, but I suggest to try to shorten it a bit (especially the paragraph related to coral restoration)

Thank you for this suggestion. We have removed text in several places, including from the paragraph on coral restoration. Omissions are clearly noted in the attached track changes document.

1) Pag. 17, Lines 410-411: use the past form for verbs (reflected, contributed..)

Sentences rewritten to read “Finally, variation in coral bleaching, growth, and survivorship among six sites in leeward Maui reflected the unique environmental history of each reef. Interactions between environmental and anthropogenic factors likely contributed to site-level variation in coral performance over time.”

2) Pag. 17, Lines 414-415. “We were…..entire populations”. Delete this sentence

Done.

**Modified references**

The following four references were added to the manuscript in response to specific requests from reviewers:

Traylor-Knowles N, Rose NH, Palumbi SR. The cell specificity of gene expression in the response to heat stress in corals. J Exp Biol. 2017;220: 1837–1845.

Bellantuono AJ, Granados-Cifuentes C, Miller DJ, Hoegh-Guldberg O, Rodriguez-Lanetty M. Coral thermal tolerance: Tuning gene expression to resist thermal stress. PLoS One. 2012;8. Available: https://doi.org/10.1371/journal.pone.0050685

Santoro EP, Borges RM, Espinoza JL, Freire M, Messias CSMA, Villela HDM, et al. Coral microbiome manipulation elicits metabolic and genetic restructuring to mitigate heat stress and evade mortality. Sci Adv. 2021;7: 19–21. doi:10.1126/sciadv.abg3088

Seveso D, Montano S, Maggioni D, Pedretti F, Orlandi I, Galli P, et al. Diel modulation of Hsp70 and Hsp60 in corals living in a shallow reef. Coral Reefs. 2018;37: 801–806.

In the course of editing the manuscript for concision, several sentences or paragraphs were removed. Through that editing process, we removed the following eight references:

- Baker AC, Starger CJ, McClanahan TR, Glynn PW. 2004. Corals’ adaptive response to climate change. Nature **430**:741–741.
- Huffmyer AS, Bean NK, Majerová E, Harris CI, Drury C. 2023. Variable intraspecific genetic diversity effects impact thermal tolerance in a reef-building coral. Coral Reefs **42**:119–129. Springer Berlin Heidelberg.

Leinbach SE, Speare KE, Strader ME. 2023. Reef habitats structure symbiotic microalgal assemblages in corals and contribute to differential heat stress responses. Coral Reefs **42**:205–217. Springer Berlin Heidelberg.

Rodríguez-Casariego JA, Mercado-Molina AE, Garcia-Souto D, Ortiz-Rivera IM, Lopes C, Baums IB, Sabat AM, Eirin-Lopez JM. 2020. Genome-Wide DNA Methylation Analysis Reveals a Conserved Epigenetic Response to Seasonal Environmental Variation in the Staghorn Coral Acropora cervicornis. Frontiers in Marine Science **7**:1–17.

Walker NS, Nestor V, Golbuu Y, Palumbi SR. 2023. Coral bleaching resistance variation is linked to differential mortality and skeletal growth during recovery. Evolutionary Applications **16**:504–517.

- Weng KC, Friedlander AM, Gajdzik L, Goodell W, Sparks RT. 2023. Decreased tourism during the COVID-19 pandemic positively affects reef fish in a high use marine protected area. PLoS ONE **18**:1–15.
- Winston M, Couch CS, Oliver TA, McCarthy OS, Speare K, Donovan MK. (*In prep*). Signs of resilience: survivorship and growth of corals in Hawaiʻi two years post-bleaching.
- Zawada KJA, Madin JS, Baird AH, Bridge TCL, Dornelas M. 2019. Morphological traits can track coral reef responses to the Anthropocene. Functional Ecology **33**:962–975.
